# Assessment of Gastric Phenotypes Using Magnifying Narrow-Band Imaging for Differentiation of Gastric Carcinomas from Adenomas

**DOI:** 10.1155/2014/274301

**Published:** 2014-10-13

**Authors:** Masaaki Kobayashi, Satoru Hashimoto, Ken Nishikura, Ken-Ichi Mizuno, Manabu Takeuchi, Yoichi Ajioka

**Affiliations:** ^1^Department of Endoscopy, Niigata University Medical and Dental Hospital, 1-757 Asahimachi-dori, Chuo-ku, Niigata 951-8520, Japan; ^2^Department of Diagnostic Pathology, Saiseikai Niigata Daini Hospital, 280-7 Teraji, Nishi-ku, Niigata 950-1104, Japan; ^3^Division of Gastroenterology, Niigata University Medical and Dental Hospital, 1-757 Asahimachi-dori, Chuo-ku, Niigata 951-8520, Japan; ^4^Division of Molecular and Diagnostic Pathology, Niigata University Graduate School of Medical and Dental Sciences, 1-757 Asahimachi-dori, Chuo-ku, Niigata 951-8510, Japan

## Abstract

*Background*. Conventional white-light endoscopy and forceps biopsy are insufficient for definitive diagnosis of gastric adenoma. Immunohistochemical studies have reported an obvious phenotypic difference between adenomas and carcinomas. We investigated the utility of narrow-band imaging with magnifying endoscopy (NBI-ME) for mucin phenotypic assessment to differentiate carcinomas from adenomas. *Methods*. NBI-ME findings were classified into A, B, and AB types, which revealed papillary, tubular pits and groove microstructures, respectively. To investigate A-B classifications retrospectively, 137 patients (155 lesions) that were diagnosed pretherapeutically with adenoma or borderline lesions by biopsy were enrolled. The mucin phenotype was analyzed immunohistochemically in the first 60 lesions. *Results*. After endoscopic submucosal dissection, A type and AB type lesions were determined histologically as carcinoma (81/82, 99%). B type lesions were adenoma (29/73, 40%) and carcinoma (44/73, 60%). A or AB type correlated to histological carcinomas (sensitivity 65%, specificity 97%, and accuracy 71%). Mucin phenotypes were gastric or gastrointestinal in A type and AB type carcinomas (31/37, 84%) and intestinal in B type adenomas and carcinomas (21/23, 91%). *Conclusions*. NBI-ME has the advantage of the assessment of mucin phenotypes in gastric carcinomas and adenomas. The proposed A-B classification is useful, especially for differentiation of gastric or gastrointestinal carcinomas from adenomas.

## 1. Introduction

Gastric adenoma (or low-grade intraepithelial neoplasia/dysplasia) is defined pathologically as a benign neoplastic tumor. Since the risk of progression from adenoma to gastric carcinoma is relatively low (approximately 0 to 9%) on long-term follow-up [1–3], most adenomas do not need aggressive clinical treatment. However, it is often difficult to discriminate between gastric adenoma and carcinoma using only conventional white-light imaging endoscopy (WLE) [4]. Endoscopic forceps biopsy is also insufficient for a definitive diagnosis and therapeutic planning in patients with gastric adenomas. Recently, several studies have shown that specimens obtained by forceps biopsy are not representative of the entire lesion [5–8]. Therefore, some authors have suggested that endoscopic resection should be considered for a precise histological diagnosis of lesions initially assessed as gastric adenomas based on forceps biopsy specimens [9, 10]. Current advances in endoscopic imaging modalities, such as narrow-band imaging with magnifying endoscopy (NBI-ME), have improved the pretherapeutic diagnostic accuracy of intraepithelial neoplasia (gastric adenoma versus carcinoma) [11–15]. However, the distinction is based on subjective judgment of the degree of irregularity in the microcapillaries and/or microstructures. Therefore, owing to the limitations of expert bias, it is not yet clear whether this method is clinically useful.

Several immunohistochemical studies have previously reported an obvious phenotypic difference between gastric adenomas and carcinomas. Most adenomas have an intestinal mucin phenotype, while many gastric carcinomas retain a gastric mucin phenotype [16]. In follow-up studies of gastric adenoma, most adenomas without malignant changes showed an intestinal phenotype, whereas some lesions that developed into carcinomas displayed a villous structure and gastric phenotype [1, 17]. The malignant lesions may initially have been gastric carcinomas with low-grade atypia that were misdiagnosed as adenoma by conventional endoscopy or biopsy examination. Mucin phenotype immunostaining of forceps biopsy samples can facilitate accurate diagnosis of borderline gastric lesions [18]. We previously described the utility of NBI-ME for the assessment of mucin phenotypes in differentiated-type mucosal gastric carcinomas [19]. NBI-ME allows visualization of the papillary and pit appearances of the surface structures, which significantly correlate with gastric and intestinal mucin phenotypes, respectively. Endoscopic differentiation of the pattern of the surface microstructure under NBI-ME may be easy to appreciate irrespective of the endoscopist's skill or experience.

The aim of the present study was to retrospectively clarify the value of NBI-ME for the diagnosis of gastric adenomas and carcinomas on the basis of mucin phenotypes. Moreover, we validated reproducibility of the proposed NBI-ME diagnosis considering the education effect for less-experienced endoscopists (LEEs).

## 2. Patients and Methods

### 2.1. Investigative Study Subjects

This study enrolled 137 patients (155 lesions) who were consecutively scheduled to undergo NBI-ME and endoscopic submucosal dissection (ESD) at our institution from April 2007 to December 2012. All patients were diagnosed before enrollment as having an adenoma or were borderline from preoperative biopsy specimens at our hospital, affiliated hospitals, or clinics. We excluded patients with a definite diagnosis of having a carcinoma on initial biopsy. Histological diagnoses of biopsy specimens, according to the classifications of the Japanese Gastric Cancer Association [20], were performed by general pathologists and specialized gastrointestinal pathologists. Our institution encourages careful follow-up of adenomas by annual endoscopic observation without further treatment. In clinical practice, however, we perform diagnostic ESD for lesions with a preoperative biopsy diagnosis of borderline or existing adenoma, but with suspicion of gastric carcinoma upon endoscopic inspection. For patients with a gastric carcinoma localized close to an adenoma, ESD is scheduled for both lesions together in one sitting. The lesions included in this investigative study were well-differentiated tubular adenocarcinomas (*n* = 125) and tubular adenomas (*n* = 30), as determined by histopathological evaluation of the samples taken at ESD.

### 2.2. Endoscopic Procedure and Diagnostic Criteria

**Figure 1:**
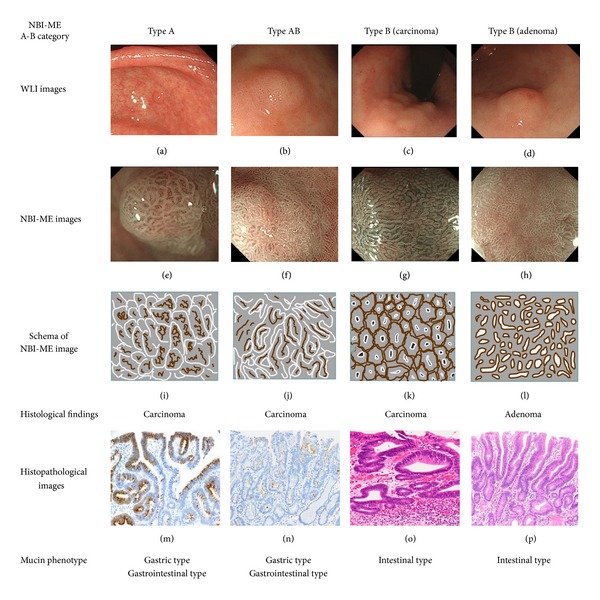
Endoscopic images of gastric carcinomas and adenomas under white-light imaging endoscopy (WLE) and narrow-band imaging with magnifying endoscopy (NBI-ME). WLE was insufficient for definitive diagnosis of carcinomas and adenomas ((a)–(d)). NBI-ME image (e) and schema (i) of A type carcinoma revealed loop-like microvessels enclosed in papillary or granular microstructures. Immunohistochemical staining demonstrated a gastric dominant phenotype. The carcinoma cells were strongly positive for MUC5AC (m). NBI-ME images of AB type carcinoma showed gyrus-like groove structures and a focal white opaque substance ((f), (j)). Immunohistochemical staining demonstrated a gastrointestinal phenotype. CD10 was expressed in the luminal surfaces of the carcinoma tubules (n). NBI-ME images of B type carcinoma ((g), (k)). The lesion showed round or tubular pits surrounded by clear mesh-form microvessels. Histological findings demonstrated intramucosal well-differentiated tubular adenocarcinoma with low-grade atypia. Despite the presence of cells with nuclear atypia similar to adenoma, this tumor presented tortuous and irregular branched glands (o). NBI-ME images of B type adenoma ((h), (l)). The lesion showed tubular pits and a diffuse white opaque substance, leading to unclear mesh-form microvessels. This lesion was histologically determined to be a tubular adenoma (p). There was a consistency of A-B classification under NBI-ME and mucin phenotypes in carcinomas and adenomas.

The instruments used in the present study were a magnifying video endoscope and an electronic endoscopic system (GIF-H260Z and EVIS LUCERA Spectrum; Olympus Medical Systems, Tokyo, Japan). As previously reported [19], NBI-ME examinations and recordings of endoscopic findings were undertaken by four highly experienced endoscopists (M.K., S.H., K.M., and M.T.). The structural enhancement level was set at B8 for the NBI mode. Before NBI-ME examinations, the gross findings, such as lesion color and central concavities, were evaluated by WLE (Figures 1(a)–1(d)) and chromoendoscopy without magnification.

As previously reported [19], NBI-ME findings were classified into A, B, or AB types. A type lesions showed loop-like microvessels enclosed in papillary or granular microstructures (Figures 1(e) and 1(i)). The microvascular architecture ran irregularly and tortuously but not over the granular structure. The mucosal surface structure had uniform or heterogeneous papillae, which appeared to be bordered with a “white zone,” as noted by Yagi et al. [21]. B type lesions showed round or tubular pits surrounded by mesh-form microvessels. Lesions that showed gyrus-like groove structures or combined findings of A type and B type lesions were designated as the AB type (Figures 1(f) and 1(j)). The NBI-ME diagnosis was determined retrospectively by a single, highly experienced endoscopist (M.K.) without pathological information.

### 2.3. Histopathological Evaluation

Histopathological evaluation of the samples taken at ESD was performed by an expert gastrointestinal pathologist (K.N.) independently of the endoscopist. Histological classification of gastric adenoma and carcinoma was based on the Japanese Gastric Cancer Association [18] and World Health Organization (WHO) classifications [22]. Carcinomas with low-grade atypia were diagnosed by the presence of papillary projections and/or irregular branched glands, even if they had cells with nuclear atypia similar to adenomas (Figures 1(o) and 1(p)). When the lesion contained varied histological atypia, the most advanced pathological finding was accepted for each lesion. Mucin phenotypes were classified into gastric, intestinal, gastrointestinal, and null types by the combination of immunohistochemical markers for gastric mucin, that is, MUC5AC (Figure 1(m)) and MUC6 and markers for intestinal mucin, that is, MUC2 and CD10 (Figure 1(n)). Immunohistochemistry and evaluation of mucin phenotypes were carried out on the first 60 lesions, as previously described [19, 23].

### 2.4. Validation Study with LEEs. 

As image evaluators, 10 LEEs participated in the study. All were trainees whose training periods in gastrointestinal endoscopy were less than 2 years. To validate the improvement in diagnostic accuracy of LEEs, we used all 30 gastric adenomas from the investigative study. In addition, as a control group, 30 carcinomas were randomly selected from the 125 carcinomas and were matched in size and treatment year to the adenoma group. Four endoscopic pictures of plain and indigo carmine dye images for conventional WLE and high-quality moderate- and high-magnification images for NBI-ME were selected for each lesion and displayed in JPEG format. Before and after the lecture, the NBI-ME and WLE image catalogs of the 60 lesions were displayed independently of each other in randomized order, and the evaluators indicated their diagnoses as either adenomas or carcinomas based on the NBI-ME and WLE images, respectively. Clinical and pathological information about the lesions, including the number of adenomas and carcinomas, were not disclosed to any of the evaluators, and discussions were not permitted among the evaluators individually.

The 1-hour training lecture was delivered by a single, highly experienced endoscopist (M.K.). This lecture described the A-B classification system of NBI-ME for differentiation of carcinoma from adenoma using an atlas of endoscopic images that did not include the study lesions. A type and AB type lesions were regarded as carcinomas on the basis of NBI-ME observations. B type lesions were further subclassified into B-carcinoma (B-ca) and B-adenoma (B-ad) types in the validation study. B-ca type lesions showed clear mesh-form microvessels and dense small pits or polymorphic pits (Figures 1(g) and 1(k)). B-ad type lesions showed unclear or faint mesh-form microvessels and uniform tubular pits (Figures 1(h) and 1(l)). According to the vascular and surface classification proposed by Yao et al. [24], B-ca type findings had an irregular microvascular pattern (open/closed-loop) plus an irregular microsurface pattern (tubular). B-ad type findings had mainly an unclear or absent microvascular pattern (obscured by a white opaque substance [25]) plus a regular microsurface pattern. Lesions that showed combined or confused findings of B-ca and B-ad types were declared as B-ca type. B-ca and B-ad type lesions were diagnosed as carcinomas and adenomas, respectively. In WLE diagnosis, larger size, reddish coloration, and the central concavity were recognized as suspicion of carcinoma according to the past reports [4, 13–15]. The study protocol was reviewed and approved by the ethics committee of our institution. Written informed consent was obtained from all participants.

### 2.5. Statistical Analysis

Baseline clinicopathological data were analyzed using the Mann-Whitney *U* test for numerical data and *χ* ^2^ test or Fisher's exact probability test for categorical data. Diagnostic accuracy was assessed with reference to histological results. The correlation between NBI-ME findings and histological data was evaluated by *χ* ^2^ test. Estimates of diagnostic accuracy were calculated based on the average for each diagnostic modality and for each NBI-ME category and before and after the training lecture for the LEE group. Diagnostic accuracies were compared with the McNemar test. Statistical analyses were performed using IBM SPSS Statistics version 21 software (IBM Japan Inc., Tokyo, Japan). Values of *P* < 0.05 were considered significant.

## 3. Results

### 3.1. Baseline Characteristics of Patients and Lesions

Clinicopathological data of the investigative study are summarized in Table 1. No significant differences were observed between carcinoma and adenoma groups, with the exception of size of lesion and morphological change on follow-up endoscopic examination. In the carcinoma group, among 44 lesions in which follow-up durations were one year or longer (mean 3.5 years), 18 (40.1%) increased in size or height. Conversely, all adenomas (*n* = 12) remained macroscopically stable over the average follow-up period of 3.7 years.

### 3.2. NBI-ME Diagnoses and Mucin Phenotypes in the Investigative Study

**Figure 2:**
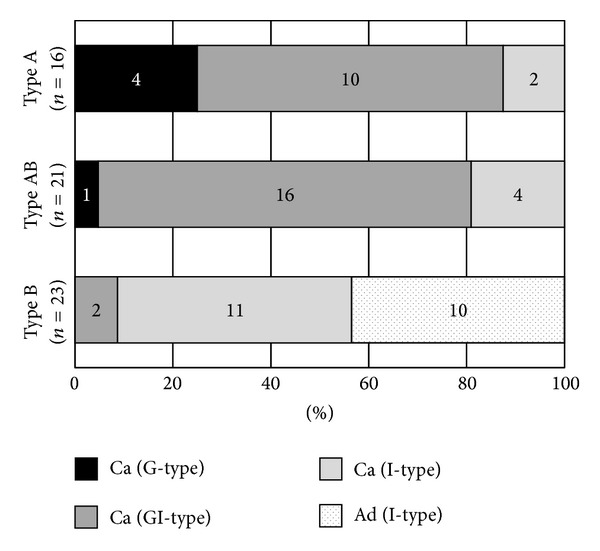
Proportion of mucin phenotypes and NBI-ME findings in gastric carcinomas (Ca) and adenomas (Ad). NBI-ME findings were determined by the A-B classification. The mucin phenotypes were classified into gastric (G), intestinal (I), and gastrointestinal (GI) by immunohistochemical markers. The correlation was significant (*P* < 0.001) among A-B categories.

NBI-ME characterized 125 carcinomas as A (*n* = 24, 19.2%), AB (*n* = 57, 45.6%), or B (*n* = 44, 35.2%) type lesions and 29 (96.7%) of the 30 adenomas as B type lesions (Table 2). In the retrospective evaluation, a NBI-ME diagnosis of A or AB type correlated with a histological diagnosis of carcinoma with a sensitivity of 64.8% (95% CI, 61.9–65.5%), specificity of 96.7% (95% CI, 84.5–99.4%), and an accuracy of 71.0% (95% CI, 66.3–72.0%). Among the first 60 lesions, the mucin phenotypes of A type or AB type carcinomas were mainly gastric or gastrointestinal types (31/37, 83.8%) and were intestinal-type in B type carcinomas (11/13, 84.6%) and B type adenomas (10/10, 100.0%). The association with histological data was significant (*P* < 0.001) among A-B categories (Figure 2).

### 3.3. Diagnostic Accuracy Based on NBI-ME Category in the Validation Study

The overall diagnostic accuracies using NBI-ME were higher than those using WLE for the 60 lesions. Even in the pretraining LEE group, the accuracy of NBI-ME (0.63; 95% CI 0.60–0.66) was significantly (*P* = 0.001) higher than that of WLE (0.57; 95% CI, 0.54–0.60). Diagnostic accuracies varied between the NBI-ME categories determined in the investigative study (Table 3). In A type and AB type lesions, the diagnostic accuracies using NBI-ME significantly improved in the LEE group after the training lecture. However, there was no improvement after the training lecture in B type lesions.

## 4. Discussion

The present study confirmed the efficacy of NBI-ME for detection of gastric phenotypes according to the proposed A-B classification system (Figure 1). NBI-ME has the advantage of visualizing the papillary or groove microstructure, which are features of carcinomas with gastric or gastrointestinal phenotypes and were identifiable by the LEEs. Even if a gastric lesion is small and diagnosed as adenoma or borderline by forceps biopsy, we recommend clinical treatment to A type and AB type lesions.

Recently, prospective trials have corroborated the diagnostic yield of NBI-ME for lesions of the stomach [26, 27]. In these studies, target lesions were limited to depressed or flat-type cancers. The discrimination of cancers from benign lesions, such as erosion, can be made on the basis of microvascular and/or microstructural irregularity. However, it is hard to recognize the difference in irregularity between carcinomas and adenomas according to their histological grade of atypia. Several authors have reported that NBI-ME appeared to be useful in differentiating carcinomas and adenomas in elevated lesions [11–15, 28]. However, these studies involved retrospective analysis by expert endoscopists on the basis of microvascular and/or microstructural irregularity. Therefore, in the present study, to clarify the value of NBI-ME for the diagnosis of carcinomas and adenomas, we first evaluated our proposed NBI-ME differentiation on the basis of surface structure and mucin phenotypes. Next, we validated the diagnostic accuracy in the LEE group before and after a training lecture about the proposed NBI-ME differentiation.

We previously confirmed a significant correlation between mucin phenotypes and NBI-ME findings, which suggested that mucin phenotypes are involved in the morphogenic differences of surface glandular structures in differentiated-type carcinomas [19]. The papillary and pit appearances under NBI-ME correlated with gastric and intestinal mucin phenotypes, respectively. Gastrointestinal-type lesions often showed a combined or intermediate pattern, such as gyrus-like groove structures. These correlations were confirmed in the lesions included in the present study, which included carcinomas diagnosed as adenomas or borderline lesions in preoperative biopsy specimens. All the adenomas in this study were intestinal-type neoplasms, and most of them had a pit appearance. According to the proposed A-B classification, none of the adenomas were classified as A type or AB type, which retained a gastric phenotype. A recent study also reported that numerous cryptal openings visualized with NBI-ME were a specific feature of gastric adenomas compared with carcinomas [29]. Although most adenomas showed an intestinal phenotype, case series of pyloric gland-type adenomas have been previously reported [30]. Because they are rare and have a high malignant potential, adenomas with gastric phenotypes should be treated as a low-grade malignancy.

The papillary or gyrus-like groove microstructure under NBI-ME was thought to be easily distinguishable by the LEE group. As expected, the diagnostic accuracies of LEEs for A type and AB type lesions improved after the training lecture. In the investigative study, 81 (65%) of 125 carcinomas, which were assessed as gastric adenoma or borderline lesions by forceps biopsy, were A type or AB type under NBI-ME. Therefore, even for LEEs, more than half of these lesions could be correctly diagnosed as carcinomas.

In contrast to A type and AB type lesions, the diagnostic accuracy of the LEE group for B type lesions did not improve after the training lecture. Endoscopic differentiation between B-ca type and B-ad type lesions is similar to the vascular and surface classification proposed by Yao et al., which is based on the degree of irregularity in the microcapillaries and/or microstructures. Accurate judgment of these findings may require experience and practice. Moreover, the histological differences between B-ca type and B-ad type lesions included nuclear atypism or glandular distortion in the deeper mucosal layer, which were hardly observed under NBI-ME. There was a limitation to differentiation of intestinal-type carcinomas from adenomas by NBI-ME. However, intestinal-type carcinomas with low-grade atypia resemble adenomas in genetic alterations and biological behaviors [31–33]. Thus, they may remain within the mucosal layer for a long time without invasion, growing slowly, similar to adenomas. In B type lesions (*n* = 26) assessed as adenomas or borderline lesions in biopsy and followed up endoscopically, we could not find enlargement, except for one B-ca type carcinoma (data not shown). Therefore, when there is a diagnostic discrepancy between histopathology of biopsy specimens and NBI-ME, only follow-up without treatment is recommended until there is endoscopic evidence of enlargement of the lesion or distinct histological evidence of a carcinoma.

There were several limitations to the present study. First, study subjects were limited to resected specimens and this caused selection bias. The number of adenomas (*n* = 30) was relatively fewer than that of carcinomas (*n* = 125), and this study did not include differentiation between neoplastic and nonneoplastic lesions. Second, it was conducted using selected endoscopic images of high quality taken by highly experienced endoscopists. Real-time diagnostic studies are required to ascertain the value of the A-B classification system under NBI-ME, especially for LEEs because this study excluded the NBI-ME technique from consideration.

## 5. Conclusions

Identification of mucin phenotype can facilitate accurate diagnosis of gastric adenomas or borderline lesions. Most adenomas have an intestinal mucin phenotype, while many carcinomas retain a gastric mucin phenotype. The proposed A-B classification system under NBI-ME is a promising approach for identification of gastric- or gastrointestinal-type carcinomas with acceptable accuracy even by LEEs, allowing stricter selection for endoscopic treatment.

## Figures and Tables

**Table 1:** Baseline clinicopathological characteristics of patients and lesions.

	Carcinoma group (113 patients, 125 lesions)	Adenoma group (29 patients, 30 lesions)
Age, years	73.0 (23–88)	72.0 (53–85)
Sex		
Male/female	77/36	22/7
Size, mm∗	13.0 (2–45)	10.0 (3–30)
<20 mm/≥20 mm	96/29	26/4
Location in the stomach		
Upper/middle/lower	18/53/54	6/10/14
Macroscopic type		
Elevated/flat or depressed	112/13	25/5
Color		
Reddish/discolored	15/110	0/30
Follow-up examination		
Yes/no	44/81	12/18
Morphological change^†^		
Yes/no	18/26	0/12

Data are expressed as number or median (range).

*P = 0.01, ^†^ *P* < 0.001.

**Table 2:** NBI-ME classifications in gastric carcinoma and adenoma groups.

NBI-ME A-B category	Carcinoma group (*n* = 125)	Adenoma group (*n* = 30)
Type A	24 (19%)	0
Type AB	57 (46%)	1 (3%)
Type B	44 (35%)	29 (97%)

Data are expressed as number (%).

NBI-ME, narrow-band imaging with magnified endoscopy.

**Table 3:** Diagnostic accuracy of less-experienced endoscopists.

NBI-ME A-B category	Pretraining accuracy (95% CI)	Posttraining accuracy (95% CI)	*P* value
Type A (*n* = 8)	0.75 (0.59–0.91)	0.93 (0.88–0.97)	0.003
Type AB (*n* = 14)	0.65 (0.46–0.84)	0.79 (0.67–0.90)	0.005
Type B (*n* = 38)	0.62 (0.41–0.82)	0.53 (0.34–0.72)	0.034

NBI-ME, narrow-band imaging with magnified endoscopy; CI, confidence interval.

## References

[1] Kolodziejczyk P, Yao T, Oya M (1994). Long-term follow-up study of patients with gastric adenomas with malignant transformation. An immunohistochemical and histochemical analysis. *Cancer*.

[2] Rugge M, Cassaro M, Di Mario F (2003). The long term outcome of gastric non-invasive neoplasia. *Gut*.

[3] Yamada H, Ikegami M, Shimoda T, Takagi N, Maruyama M (2004). Long-term follow-up study of gastric adenoma/dysplasia. *Endoscopy*.

[4] Jung MK, Jeon SW, Park SY (2008). Endoscopic characteristics of gastric adenomas suggesting carcinomatous transformation. *Surgical Endoscopy and Other Interventional Techniques*.

[5] Muehldorfer SM, Stolte M, Martus P, Hahn EG, Ell C (2002). Diagnostic accuracy of forceps biopsy versus polypectomy for gastric polyps: a prospective multicentre study. *Gut*.

[6] Kim YJ, Park JC, Kim J-H (2010). Histologic diagnosis based on forceps biopsy is not adequate for determining endoscopic treatment of gastric adenomatous lesions. *Endoscopy*.

[7] Takao M, Kakushima N, Takizawa K (2012). Discrepancies in histologic diagnoses of early gastric cancer between biopsy and endoscopic mucosal resection specimens. *Gastric Cancer*.

[8] Kasuga A, Yamamoto Y, Fujisaki J (2012). Clinical characterization of gastric lesions initially diagnosed as low-grade adenomas on forceps biopsy. *Digestive Endoscopy*.

[9] Cho S-J, Choi IJ, Kim CG (2011). Risk of high-grade dysplasia or carcinoma in gastric biopsy-proven low-grade dysplasia: an analysis using the Vienna classification. *Endoscopy*.

[10] Choi CW, Kang DH, Kim HW, Park SB, Kim S, Cho M (2012). Endoscopic submucosal dissection as a treatment for gastric adenomatous polyps: predictive factors for early gastric cancer. *Scandinavian Journal of Gastroenterology*.

[11] Nakamura M, Shibata T, Tahara T (2010). The usefulness of magnifying endoscopy with narrow-band imaging to distinguish carcinoma in flat elevated lesions in the stomach diagnosed as adenoma by using biopsy samples. *Gastrointestinal Endoscopy*.

[12] Nonaka K, Arai S, Ban S (2011). Prospective study of the evaluation of the usefulness of tumor typing by narrow band imaging for the differential diagnosis of gastric adenoma and well-differentiated adenocarcinoma. *Digestive Endoscopy*.

[13] Miwa K, Doyama H, Ito R (2012). Can magnifying endoscopy with narrow band imaging be useful for low grade adenomas in preoperative biopsy specimens?. *Gastric Cancer*.

[14] Tsuji Y, Ohata K, Sekiguchi M (2012). Magnifying endoscopy with narrow-band imaging helps determine the management of gastric adenomas. *Gastric Cancer*.

[15] Maki S, Yao K, Nagahama T (2013). Magnifying endoscopy with narrow-band imaging is useful in the differential diagnosis between low-grade adenoma and early cancer of superficial elevated gastric lesions. *Gastric Cancer*.

[16] Tsukashita S, Kushima R, Bamba M, Sugihara H, Hattori T (2001). MUC gene expression and histogenesis of adenocarcinoma of the stomach. *International Journal of Cancer*.

[17] Tajima Y, Yamazaki K, Makino R (2006). Gastric and intestinal phenotypic marker expression in early differentiated-type tumors of the stomach: clinicopathologic significance and genetic background. *Clinical Cancer Research*.

[18] Minematsu H, Saito Y, Kakinoki R, Andoh A, Kushima R, Fujiyama Y (2006). Evaluation of mucin expression patterns in gastric borderline (group III) lesions. *Journal of Gastroenterology*.

[19] Kobayashi M, Takeuchi M, Ajioka Y (2011). Mucin phenotype and narrow-band imaging with magnifying endoscopy for differentiated-type mucosal gastric cancer. *Journal of Gastroenterology*.

[20] Japanese Gastric Cancer Association (2011). Japanese classification of gastric carcinoma: 3rd English edition. *Gastric Cancer*.

[21] Yagi K, Nozawa Y, Endou S, Nakamura A (2012). Diagnosis of early gastric cancer by magnifying endoscopy with NBI from viewpoint of histological imaging: Mucosal patterning in terms of white zone visibility and its relationship to histology. *Diagnostic and Therapeutic Endoscopy*.

[22] Lauwers GY, Carneiro F, Graham DY, Bosman FT, Carneiro F, Hruban RH, Theise ND (2010). Gastric carcinoma. *WHO Classification of Tumours of the Digestive System*.

[23] Shiroshita H, Watanabe H, Ajioka Y, Watanabe G, Nishikura K, Kitano S (2004). Re-evaluation of mucin phenotypes of gastric minute well-differentiated-type adenocarcinomas using a series of HGM, MUC5AC, MUC6, M-GGMC, MUC2 and CD10 stains. *Pathology International*.

[24] Yao K, Anagnostopoulos GK, Ragunath K (2009). Magnifying endoscopy for diagnosing and delineating early gastric cancer. *Endoscopy*.

[25] Yao K, Iwashita A, Tanabe H (2008). White opaque substance within superficial elevated gastric neoplasia as visualized by magnification endoscopy with narrow-band imaging: a new optical sign for differentiating between adenoma and carcinoma. *Gastrointestinal Endoscopy*.

[26] Kato M, Kaise M, Yonezawa J (2010). Magnifying endoscopy with narrow-band imaging achieves superior accuracy in the differential diagnosis of superficial gastric lesions identified with white-light endoscopy: a prospective study. *Gastrointestinal Endoscopy*.

[27] Ezoe Y, Muto M, Uedo N (2011). Magnifying narrowband imaging is more accurate than conventional white-light imaging in diagnosis of gastric mucosal cancer. *Gastroenterology*.

[28] Mochizuki Y, Saito Y, Kobori A (2012). Magnifying endoscopy with narrow-band imaging in the differential diagnosis of gastric adenoma and carcinoma and identification of a simple indicator. *Journal of Gastrointestinal and Liver Diseases*.

[29] Kanesaka T, Sekikawa A, Tsumura T (2014). Dense-type crypt opening seen on magnifying endoscopy with narrow-band imaging is a feature of gastric adenoma. *Digestive Endoscopy*.

[30] Vieth M, Kushima R, Mukaisho K-I, Sakai R, Kasami T, Hattori T (2010). Immunohistochemical analysis of pyloric gland adenomas using a series of Mucin 2, Mucin 5AC, Mucin 6, CD10, Ki67 and p53. *Virchows Archiv*.

[31] Shibata N, Watari J, Fujiya M, Tanno S, Saitoh Y, Kohgo Y (2003). Cell kinetics and genetic instabilities in differentiated type early gastric cancers with different mucin phenotype. *Human Pathology*.

[32] Sugai T, Inomata M, Uesugi N (2004). Analysis of mucin, p53 protein and Ki-67 expression in gastric differentiated-type intramucosal neoplastic lesions obtained from endoscopic mucosal resection samples: a proposal for a new classification of intramucosal neoplastic lesions based on nuclear atypia. *Pathology International*.

[33] Yamazaki K, Tajima Y, Makino R (2006). Tumor differentiation phenotype in gastric differentiated-type tumors and its relation to tumor invasion and genetic alterations. *World Journal of Gastroenterology*.

